# Reaction Engineering for Asymmetric *R*‐/*S*‐PAC Synthesis With Ephedrine or Pseudoephedrine Dehydrogenase in Pickering Emulsion

**DOI:** 10.1002/elsc.202400069

**Published:** 2025-01-06

**Authors:** Reynaldo Jr. Carubio, Bao‐Hsiang Wang, Marion B. Ansorge‐Schumacher

**Affiliations:** ^1^ Chair of Molecular Biotechnology Dresden University of Technology Dresden Germany

**Keywords:** bioactive Pickering emulsions, design of experiment, enzyme cascade, microbioreactor, phenylacetylcarbinol

## Abstract

The synthesis of enantiopure α‐hydroxy ketones, particularly *R*‐ and *S*‐phenylacetylcarbinol (PAC), represents an important process in the pharmaceutical industry, serving as a pivotal step in the production of drugs. Recently, two novel enzymes, ephedrine dehydrogenase (EDH) and pseudoephedrine dehydrogenase (PseDH), have been described. These enzymes enable the specific reduction of 1‐phenyl‐1,2‐propanedione (PPD) to *R*‐PAC and *S*‐PAC, respectively. In this study, we transferred these enzymes into Pickering emulsions, which is an attractive reaction set‐up for large‐scale synthesis. The bioactive w/o Pickering emulsion (bioactive Pickering emulsion [BioPE]), in which methyl *tert*‐butyl ether served as the continuous phase, was stabilized by silica nanoparticles. Formate dehydrogenase from *Rhodococcus jostii* was utilized for cofactor regeneration. Given the considerable complexity of the BioPE, this reaction system underwent a first‐time application of design of experiment (DOE) for systematic engineering. A definitive screening design was employed to identify significant factors affecting space‐time yield (STY) and conversion. Response surface methodology was used to optimize the conditions, resulting in the observation of a high STY of 4.2 g L⁻¹ h⁻¹ and a conversion of 83.2% for BioPE with EDH, and an STY of 4.4 g L⁻¹ h⁻¹ and a conversion of 64.5% for BioPE with PseDH.

**Abbreviations**
BioPE: bioactive Pickering emulsion
cP: continuous phase
dP: dispersed phase
DSD: definitive screening design
EDH: ephedrine dehydrogenase
MTBE: methyl *tert*‐butyl ether
NADH: nicotinamide adenine dinucleotide
PAC: phenylacetylcarbinol
PPD: 1‐phenyl‐1,2‐propanedione
PseDH: pseudoephedrine dehydrogenase
RjFDH: *Rhodococcus jostii* formate dehydrogenase
STY: space‐time yield

## 1 Introduction

The synthesis of enantiopure α‐hydroxy ketones, particularly *R*‐ and *S*‐phenylacetylcarbinol (PAC), is of great importance in the pharmaceutical industry. They serve as essential intermediates in the production of various valuable fine chemicals and pharmaceuticals [1]. *R*‐PAC and *S*‐PAC are precursors to the synthesis of ephedrine, pseudoephedrine, and norephedrine, which are used as key ingredients in numerous valuable medications, including decongestants, antiasthmatics, and bronchodilators [2].

Currently, *R*‐PAC in particular is produced on a large scale due to the greater importance of its pharmaceutical derivatives compared to those of *S*‐PAC. The process is primarily dependent on enzymatic C–C coupling during the fermentation of the yeast *Saccharomyces cerevisiae* in the presence of benzaldehyde [3]. This method offers an economical advantage due to the low cost of the starting materials and reagents. However, various alcohol dehydrogenases in the yeast cells also generate unwanted by‐products, such as benzyl alcohol and 1‐phenyl‐1,2‐propanediol [4, 5]. The objective of enhancing the production of enantiopure *R*‐PAC has been pursued through the identification of pyruvate decarboxylase (PDC) as the primary enzyme responsible for PAC production in yeast and its subsequent purification [6, 7, 8]. As an alternative approach, related enzymes from the same class have been employed for the reaction. These include PDC from *Zymomonas mobilis*, provided in a recombinant *Escherichia coli* whole cell reaction system (*R*‐PAC yield between 40% and 70%) and isolated keto acid decarboxylase (KdcA) from *Lactococcus lactis* (*R*‐PAC yield of 40%) [9, 10]. However, the generation of by‐products resulting from nonspecific C─C bond formation from the initial carbonyls, such as acetoin, benzoin, and 2‐hydroxypropiophenone (HPP), represents a significant and persistent challenge.

In regard to the production of *S* [11], namely the dynamic kinetic resolution (DKR) of racemic PAC, which has yielded *S*‐PAC with an enantiomeric excess of 81% [12]. The system employed an immobilized lipase from *Pseudomonas stutzeri* for the enantiospecific transesterification of *S*‐PAC and a zirconium‐based heterogeneous catalyst, designated as Zr‐TUD‐1 (TU Delft, The Netherlands), for the racemization of the residual *R*‐PAC. However, as the final product of the reaction was the *S*‐PAC‐butyrate, a subsequent work‐up was required to release *S*‐PAC, resulting in a low product yield of only 38%. Furthermore, a considerable quantity of the diketone corresponding to PAC, 1‐phenyl‐1,2‐propanedione (PPD), was identified as a by‐product from the racemization process.

Recently, two oxidoreductases with the distinct capacity to catalyze the regio‐ and enantiospecific reduction of PPD into *R*‐PAC or *S*‐PAC, respectively, have been described [13], thereby facilitating novel avenues for both *R*‐ and *S*‐PAC synthesis. The enzymes, ephedrine dehydrogenase (EDH) and pseudoephedrine dehydrogenase (PseDH) from *Arthrobacter* sp. TS‐15, achieve complete conversion of PPD and 99% enantiomeric excess without the generation of by‐products [14]. However, a significant limitation of utilizing these enzymes, and most other native enzyme catalysts, is their requirement for an aqueous environment to maintain activity [15, 16]. This presents a challenge for efficient conversion and synthesis of hydrophobic organic compounds such as PPD and PAC.

One potential avenue for addressing this challenge is the introduction of EDH and PseDH to a Pickering emulsion (PE), a type of emulsion stabilized by solid particles adsorbed at the interface between two immiscible liquid phases [17]. It allows enzymes to be located in the dispersed aqueous phase, while substrates and products are dissolved in a water‐immiscible continuous phase (cP). Bioactive Pickering emulsions (BioPEs) are widely acknowledged for their ability to streamline product separation and enhance enzymatic interfacial activation [19, 20, 21, 22]. The distinctive quality of BioPE lies in the exceptional stability of the droplets forming the dispersed phase (dP), which allows it to facilitate novel microenvironmental reactions [23]. Moreover, BioPE prevents enzyme deactivation and benefits from a high interfacial area, which results in increased reaction yields [18]. The simplicity of sequential modification of solid particles allows for the selective, oriented, and spatial immobilization of multiple biocatalysts, rendering BioPE a more attractive option for enzyme cascade reactions than conventional systems [24, 25].

**SCHEME 1:**
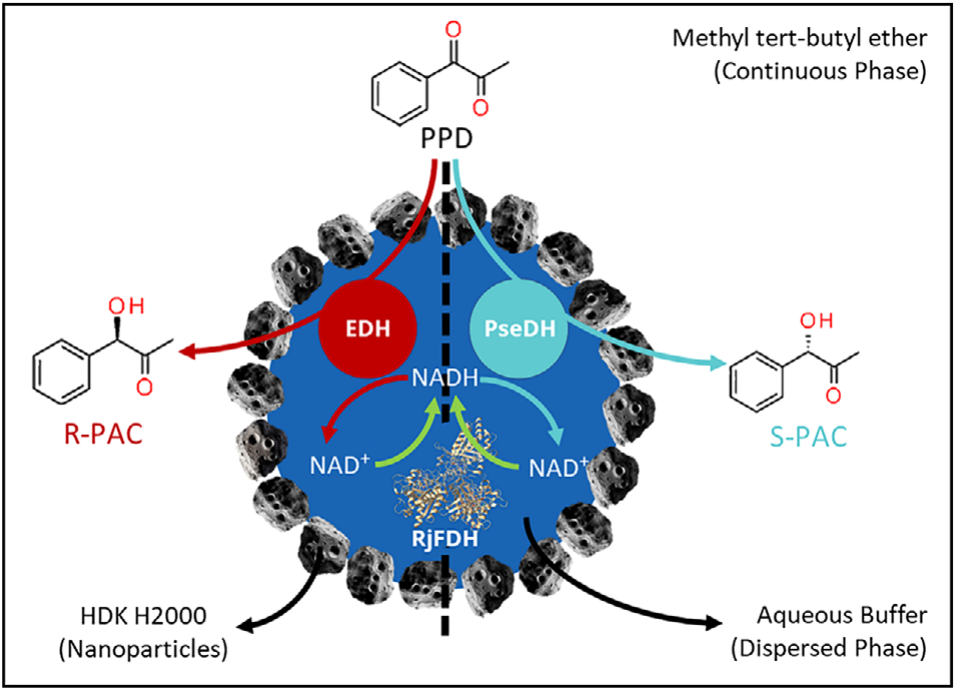
Enzyme cascades for *R*‐/*S*‐phenylacetylcarbinol (PAC) synthesis in Pickering emulsion.

In this study, we developed, optimized, and evaluated a water‐in‐oil (w/o) BioPE for the synthesis of *R*‐ or *S*‐PAC (Scheme 1). The aqueous enzyme solution, comprising either EDH for *R*‐PAC production or PseDH for *S*‐PAC production, served as the dP. Both enzymes were coupled with formate dehydrogenase from *Rhodococcus jostii* (*Rhodococcus jostii* formate dehydrogenase [RjFDH]) for cofactor regeneration. This enzyme was selected due to its superior thermal stability and catalytic efficiency in nicotinamide adenine dinucleotide (NADH) regeneration compared to other commonly used enzymes such as formate dehydrogenase from *Candida boidinii* [14, 26]. The cP was methyl *tert*‐butyl ether (MTBE) due to its minimal environmental impact, as previously documented [27]. Additionally, it has been demonstrated that MTBE may positively affect the enzyme stability in a biphasic biocatalytic system [28]. The nanoparticle utilized was the silicate material HDK H2000, which has already been shown to stabilize BioPE [29]. Given the considerable complexity of the BioPE system, we employed a design of experiment (DOE) for the first time on this type of reaction system to identify the significant factors affecting the space‐time yield (STY) and conversion efficiency. Optimization of conditions was conducted using response surface methodology.

## 2 Materials and Methods

### 2.1 Materials and Chemicals

MTBE was purchased from Thermo Fisher Scientific (Breda, Netherlands). PPD was purchased from Thermo Fisher Scientific (MA, USA). Potassium formate, potassium diphosphate, dipotassium phosphate, and NADH were purchased from Carl Roth GmbH + Co. KG (Karlsruhe, Germany). HDK H2000 hydrophobic silica nanoparticles were provided by Wacker Chemie AG (Burghausen, Germany). Milli‐Q water was used for all experiments.

The enzymes EDH, PseDH, and RjFDH were produced and purified according to the protocols described in the original publications [14, 26]. The obtained specific activities of EDH and PseDH for PPD reduction are 78.4 and 5.8 U mg^−1^, respectively. The obtained specific activity of RjFDH for potassium formate oxidation is 5.7 U mg^−1^. The enzyme activity unit, U, is defined as the amount of enzyme that converts 1 µmol of substrate per minute under the assay conditions.

### 2.2 Preparation of w/o Pickering Emulsions

cP and dP were prepared separately. For cP, 100 g L_dP_^−1^ (with respect to dP) of HDK H2000 nanoparticles were suspended in MTBE and then ultrasonicated for 10 min. For dP, 0.75 mmol L^−1^ NADH and 0.04 g⋅L^−1^ EDH or 0.2 g L^−1^ PsEDH were dissolved with varying concentrations of potassium formate and RjFDH in potassium phosphate buffer (pH 7.5) at the desired concentration (see Tables S1 and S2). The two phases were combined in the desired phase ratio and then subjected to homogenization at 17,400 rpm for 2 min with an UltraTurrax T18 (IKA‐Werke GmbH & Co. KG, Staufen, Germany). The total volume of Pickering emulsion prepared per experimental run was 20 mL.

### 2.3 Analytical Methods

#### 2.3.1 Gas Chromatography

Substrate and product concentrations were measured using a Shimadzu GC2010 gas chromatograph (Duisburg, Germany) equipped with a flame ionization detector and HYDRODEX GAMMA‐DiMOM column (Macherey‐Nagel, Germany), using nitrogen as the carrier gas at a flowrate of 30 mL min^−1^. Temperature program: 125°C for 25 min, increase at a rate of 15°C min^−1^ up to 190°C. Flame ionization detector at 290°C. Retention times: 7.81 min (PPD), 16.02 min (*S*‐phenylacetyl carbinol), and 18.72 min (*R*‐phenylacetyl carbinol).

#### 2.3.2 Imaging

Images of PE samples were captured using a Nikon Eclipse Ts2 inverted microscope (Düsseldorf, Germany) and analyzed manually with the NIS Elements Imaging software from Nikon Instruments Inc. (Düsseldorf, Germany). An average of 85 droplets were measured per sample.

### 2.4 Experimental Design

#### 2.4.1 Definitive Screening Design (Factor Screening)

The significances of dispersion speed, dispersion time, reaction mixing speed, nanoparticle concentration, cP ratio, phosphate buffer concentration, potassium formate concentration, RjFDH loading, and substrate concentration on STY and conversion in the BioPE were determined using definitive screening design (DSD). Considering information from literature on the creation of PE, the dispersion speed was varied from 8,000 to 17,400 rpm, the dispersion time was varied from 1 to 2 min, the speed of reaction mixing was varied from 20 to 40 rpm, the cP ratio was varied from 0.6 to 0.8, and the nanoparticle concentration was varied from 37.5 to 100 g L^−1^. To account for the optimal buffer and substrate concentrations of the enzymes, the phosphate buffer concentration was varied from 0.1 to 1.0 mol L^−1^, the potassium formate concentration was varied from 0.625 to 1.5 mol⋅L^−1^, the substrate concentration was varied from 5 to 12.5 mmol⋅L^−1^, and the RjFDH concentration was varied from 0.1 to 0.25 g⋅L^−1^. The NADH concentration was kept constant. The experimental designs are presented in the Supporting Information (Tables S1 and S2). A total of 38 experimental runs were generated using the DSD of the statistical program JMP Pro version 17 from JMP Statistical Discovery LLC (NC, USA).

#### 2.4.2 Response Surface Methodology (STY and Conversion Optimization)

The STY and conversion optimization of the reaction in the BioPE was done by subjecting the significant factors of the DSD experiment to a central composite design with six replications of the center point. A total of 32 experimental runs were generated through the use of the response surface design feature of the statistical program JMP Pro version 17. The experimental design for BioPE with EDH and PseDH, respectively, is presented in the Supplementary Information (Tables S3 and S4). The reaction temperatures were 25 and 8°C for BioPE with EDH and PseDH, respectively.

Samples were extracted at 55, 90, and 125 min for each run. The observed STY and conversion rates relative to the extraction time were imported into the JMP Pro software, where a standard least squares modeling approach was employed to identify influential factors and interactions. Those with *p* value more than 0.05 were removed, and a second‐order polynomial prediction expression was obtained. This expression indicates that only one local extremum can be modeled.

## 3 Results and Discussion

### 3.1 Significant Factors for BioPE Functionality

The BioPE employed in this study can essentially be considered as a combination of two intricate fundamental systems: the reactive enzyme cascade and the stable water‐in‐oil (w/o) emulsion. Both can interactively impact the functionality of the BioPE, as assessed in terms of substrate conversion and STY. The influence of the basic systems is contingent upon a multitude of contributing factors, including enzyme, substrate, cosubstrate, and buffer concentrations in the reactive cascade and phase ratio, speed and time of phase dispersion, nanoparticle concentration, and mixing speed in the emulsion [19, 21, 30, 31, 32, 33, 34, 35]. A number of these factors were screened for significant influences on the BioPE performance (Table 1), employing the BioPE with PseDH as the model system due to the ease of production and purification of PseDH in comparison to EDH. PseDH loading was maintained at a constant 35 mg⋅L^−1^ to optimize enzyme usage and prevent bias in STY and conversion data. Conversely, the concentration of RjFDH was varied in order to investigate the impact of the regeneration system on overall performance.

**TABLE 1:** *p* values of main effects from DSD experiments.

	*p* value
Phosphate buffer concentration	0.00000
PPD concentration	0.00004
RjFDH loading	0.00006
Potassium formate concentration	0.00015
cP ratio	0.00389
Dispersion speed	0.12676
Dispersion time	0.24003
Nanoparticle concentration	0.47671
Mixing speed	0.83533

Abbreviations: cP, continuous phase; DSD, definitive screening design; PPD, 1‐phenyl‐1,2‐propanedione; RjFDH, *Rhodococcus jostii* formate dehydrogenase.

Significant factors for BioPE functionality were identified using a DSD approach to ensure that the main effect estimates were unbiased by secondary effects [36]. The resulting *p* values are shown in Table 1.

The *p* value is a measure used to determine the probability of obtaining the observed results under the assumption that there is no true effect or relationship in the population under study [37]. Main effects with *p* value less than the threshold *p* value are considered significant. The default selected threshold *p* value was 0.05. Accordingly, significant factors for BioPE functionality were the cP ratio (cP ratio), potassium formate concentration, RjFDH loading, substrate concentration, and phosphate buffer concentration. Interestingly, with the exception of the cP ratio, these are all related to the enzyme cascade and are components of the dP, whereas factors related to the Pickering emulsion, which are generally thought to be important for the performance of reactive PE [34, 35, 38], were not significant here. The significance of the cP ratio could be explained by its correlation with the amount of dP in the Pickering emulsion: As the percentage of dP increases, assuming the droplet size remains constant, the total volume of the droplets increases, leading to an increase in the total interfacial area [32].

### 3.2 Prediction Expression for STY and Conversion

Using the identified significant factors for BioPE functionality as the independent variables, response surface methodology generated prediction expressions for STY and conversion for both the reactions with PseDH and EDH (Table 2), establishing the relationship between the respective dependent variable and the independent variables.

**TABLE 2:** Regression equations for space‐time yield (STY) and conversion of BioPE with EDH and PseDH, respectively.

Parameter	Prediction expression	*R* ^2^
STY with EDH (*R*‐PAC) (g L^−1^ h^−1^)	4.02166 − 4.23791*A* + 0.13120*B* − 0.95347*C* − 1.81359*D* + 7.68101*E* − 0.03336*F* − 0.06593A*B + 8.54815A*C + 1.66519A*D − 1.41975A*E − 0.03833B*C − 0.01971B*D − 0.01672B*E − 0.664C*D − 3.49444C*E + 0.01240C*F + 0.07778D*E + 0.005157D*F − 0.0004804*B* ^2^ − 6.42419*C* ^2^ − 5.13283*E* ^2^ + 0.00008599*F* ^2^	0.97
Conversion with EDH (%)	97.98418 − 108.6463*A* − 1.33157*B* − 42.8771*C* − 31.1559*D* + 197.2487*E* + 0.74999*F* + 0.58585A*B + 263.074A*C − 0.3641B*C − 19.5052A*D + 0.3762B*D − 22.644C*D − 74.9938A*E − 0.751083B*E − 74.5139C*E − 0.13896C*F − 0.08039D*F + 23.5967D*E + 0.0038231*B* ^2^ − 133.973*C* ^2^ − 106.318*E* ^2^ − 0.0020939*F* ^2^	0.97
STY with PseDH (*S*‐PAC) (g L^−1^ h^−1^)	4.327053 − 3.87748*A* + 0.0747884*B* + 1.77737*C* + 0.464297*D* + 2.494445*E* − 0.0540341*F* + 3.45015A*C − 0.0685273B*C − 0.369987C*D − 6.7721×10^−7^ *B* ^2^ − 2.33416*C* ^2^ − 0.332474 *D* ^2^ − 1.66965 *E* ^2^ + 0.000206675*F* ^2^	0.80
Conversion with PseDH (%)	60.4654 − 67.7676*A* − 1.26982*B* + 48.8511*C* + 12.0628*D* + 57.4341*E* + 0.616803*F* + 63.1988A*C − 0.925168B*C − 13.3063C*D + 0.0153525*B* ^2^ − 66.4361*C* ^2^ − 8.44626*D* ^2^ − 33.5553*E* ^2^ − 0.00165559*F* ^2^	0.90

*Note:* *A*, *B*, *C*, *D*, *E*, and *F* are cP ratio, substrate concentration (mM), phosphate buffer concentration (M), potassium formate concentration (M), RjFDH loading (g L^−1^), and time (min), respectively.

Abbreviations: BioPE, bioactive Pickering emulsion; cP, continuous phase; EDH, ephedrine dehydrogenase; PAC, phenylacetylcarbinol; PseDH, pseudoephedrine dehydrogenase; RjFDH, *Rhodococcus jostii* formate dehydrogenase.

The analysis of variance (ANOVA) showed that all the linear terms were significant (*p* < 0.05), while some of the quadratic and interaction terms were insignificant. For BioPE with EDH, significant interactions were identified specifically between the phase ratio and the following variables: substrate concentration, phosphate buffer concentration, potassium formate concentration, and RjFDH loading. Additionally, interactions were observed between the substrate concentration and the following variables: phosphate buffer concentration, potassium formate concentration, and RjFDH loading. The phosphate buffer concentration also interacted with the potassium formate concentration, the RjFDH loading, and time; the potassium formate concentration also interacted with RjFDH loading and with time. Significant interactions for BioPE with PseDH were found specifically between the phosphate buffer concentration and the following variables: phase ratio, substrate concentration, and potassium formate concentration.

It is noteworthy that the BioPE with PseDH exhibited a diminished number of interactions in comparison to the BioPE with EDH. This discrepancy may be attributed to the variations in reaction temperatures and factor settings for phosphate buffer and potassium formate concentrations in their respective experimental designs, which were necessary to achieve superior yields [39]. In particular, the reaction temperature for the BioPE with EDH was set at 25°C, while that for the BioPE with PseDH was set at 8°C. This modification was implemented in accordance with the finding that PseDH exhibited superior performance at 8°C compared to 25°C (see Figure S1), which reflects the considerably higher temperature optimum for the catalyzed reaction of EDH (approximately 75°C) in comparison to PseDH (35°C) [14]. Similarly, the enzymes require varying concentrations of phosphate buffer and potassium formate for optimal performance; PseDH exhibits a preference for higher concentrations of both [40].

The predictive validity of the model was confirmed by several key metrics. The *R* ^2^ values calculated for each scenario assessed the goodness‐of‐fit. The *R* ^2^ values were generally high (see Table 2), indicating that the model explains a substantial portion of the variance in the response variables. Values close to 1 suggest an excellent fit and a strong predictive capability [39]. In agreement, ANOVA yielded high *F* ratios and low *p* values. For BioPE with EDH, the STY model had an *F* ratio of 103.3 with a *p* value of 0.0001, and the conversion model had an *F* ratio of 95.0 with a *p* value of 0.0001. For BioPE with PseDH, the STY model had an *F* ratio of 25.2 with a *p* value of 0.0001, and the conversion model had an *F* ratio of 54.5 with a *p* value of 0.001. These high *F* ratios and low *p* values indicate that the models are statistically significant, demonstrating that the predictors collectively explain a significant proportion of the variance in the response variables [39]. On the other hand, in the lack of fit test the STY model exhibited an *F* ratio of 1.4 with a *p* value of 0.1996, while for the conversion model an *F* ratio of 5.1 and a *p* value of 0.0002 was found for BioPE with EDH. For BioPE with PsEDH, the STY model yielded an *F* ratio of 1.9 with a *p* value of 0.0565, and the conversion model produced an *F* ratio of 3.0 and a *p* value of 0.0032. In the lack of fit test, high *F* ratios indicate substantial discrepancies between the predicted and obtained data, whereas a high *p* value suggests that there is no evidence of a significant lack of fit, indicating that the model adequately represents the data [39]. Accordingly, the STY model for BioPE with EDH demonstrated no substantial lack of fit, whereas the conversion model exhibited a notable lack of fit, as indicated by a *p* value that was considerably below the 0.05 significance threshold. The STY model for BioPE with PseDH exhibited marginal evidence for lack of fit, though this was not statistically significant at the 0.05 level. In contrast, the conversion model for this system demonstrated a significant lack of fit, as evidenced by a *p* value that fell well below the 0.05 threshold. These results suggest that while the models perform well for predicting STY, they may need refinement to better capture conversion outcomes, as the conversion response might be influenced by factors or interactions not fully accounted for in the model, and could be more complex and sensitive to variations across the experimental space. The lack of fit analysis, based on replication at the centerpoint, primarily assesses model adequacy around the center of the experimental space, with potential limitations in detecting a lack of fit across the entire design space.

### 3.3 STY and Conversion in Optimized Setting

The independent variables that would result in the optimal STYs and conversions for the two BioPE systems, which were obtained from the gradient descent algorithm, are presented in Table 3, along with their predicted values.

**TABLE 3:** JMP‐generated values of significant factors to achieve optimal space‐time yield (STY) and conversion in BioPE.

BioPE	cP ratio	PPD concentration (mmol L^−1^)	Phosphate buffer concentration (mol L^−1^)	Potassium formate concentration (mol L^−1^)	RjFDH loading (g L^−1^)	Predicted STY (g L^−1^ h^−1^)	Predicted conversion (%)
EDH	0.525	26.5	0.04	0.25	0.65	4.2	81.7
PseDH	0.525	37.5	0.23	0.1	0.80	4.2	60.9

Abbreviations: BioPE, bioactive Pickering emulsion; cP, continuous phase; EDH, ephedrine dehydrogenase; PseDH, pseudoephedrine dehydrogenase; RjFDH, *Rhodococcus jostii* formate dehydrogenase.

**FIGURE 1:**
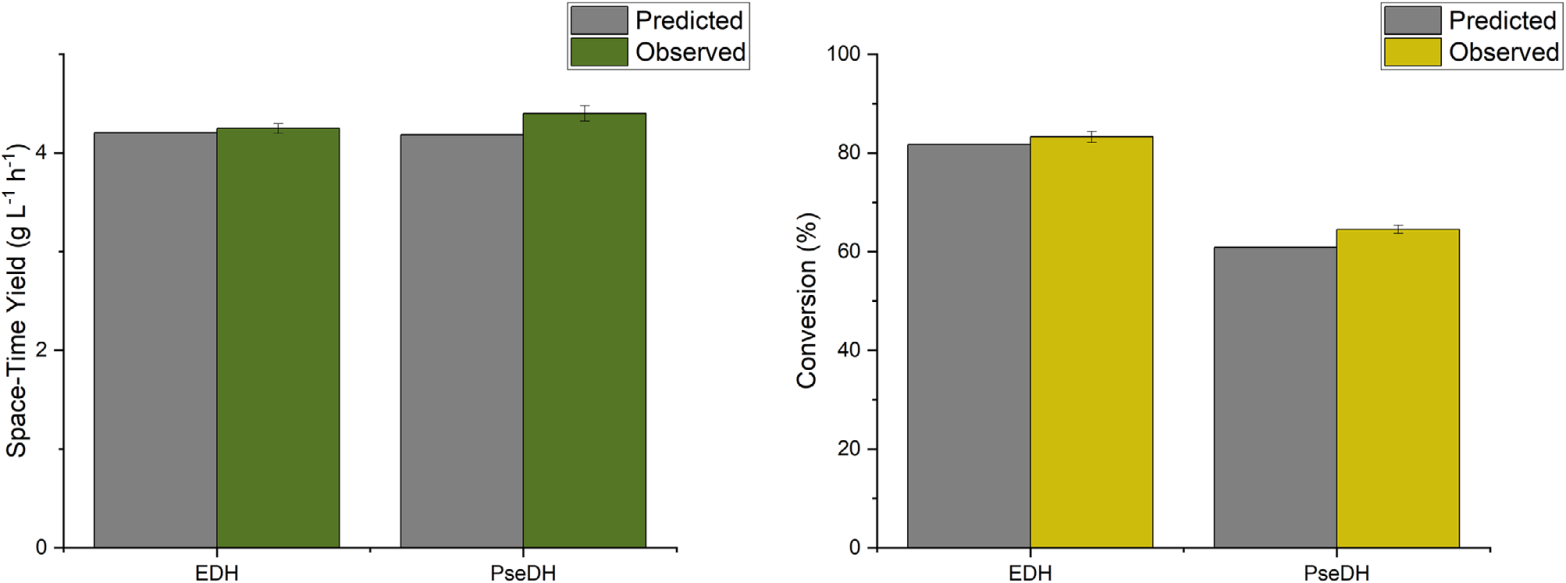
Comparison of the predicted and observed optimal STY and conversion of BioPE with EDH and PseDH, respectively. BioPE, bioactive Pickering emulsion; EDH, ephedrine dehydrogenase; PseDH, pseudoephedrine dehydrogenase; STY, space‐time yield.

In the case of BioPE with EDH, the STY measured at the parameter setting for optimal functionality was 4.2 g*_R_*_‐PAC_ L^−1^ h^−1^, with a conversion at 83.2%. For BioPE with PseDH, the STY was found to be in the same range, at 4.4 g*_S_*_‐PAC_ L^−1^ h^−1^, while conversion was 64.5%. The results were in close alignment with the predicted values (Figure 1). The approximation error for the STY was 1% and 5%, and for the conversion was 1% and 6% for BioPE with EDH and PsEDH, respectively, demonstrating the robust predictive performance of the models.

Compared to the STY and conversion of alternative PAC‐production systems that can be calculated from the literature, the performance of both BioPE with EDH and PseDH in the optimized system is very good. In a patent describing the production of *R*‐PAC using isolated PDC from *Z. mobilis* in a two‐phase system [28], a maximum *R*‐PAC production of 117.5 g L⁻¹ was described after 72 h, corresponding to an STY of 1.6 g L⁻¹ h⁻¹. This is only 38% of the STY observed with EDH in BioPE. With an engineered variant of PDC from *Acetobacter pasteurianus*, 48 g L⁻¹ *S*‐PAC were produced within 10 h, corresponding to an STY of 4.8 g L⁻¹ h⁻¹ [41], which is comparable with PseDH in BioPE. Likewise, an STY of 4.5 g L⁻¹ h⁻¹ *R*‐PAC and conversion of 84%, which is in the same range as EDH, has been described for whole cells of *S. cerevisiae* using supercritical fluids or liquefied gas instead of an aqueous phase [42]. Only the chemo‐enzymatic DKR with immobilized lipases achieved a considerably higher STY of 12.6 g L⁻¹ h⁻¹ *S*‐PAC so far [11]. Notably, however, all alternative methods used much higher catalyst concentrations than in our study (with EDH limited to 0.04 g L⁻¹ and PseDH to 0.2 g L⁻¹). For DKR, for example, 20 g L⁻¹ immobilized lipase and additional 60 g L⁻¹ heterogeneous chemo‐catalyst were used [11].

### 3.4 Enzyme and PE Stability in Optimized Setting

**FIGURE 2:**
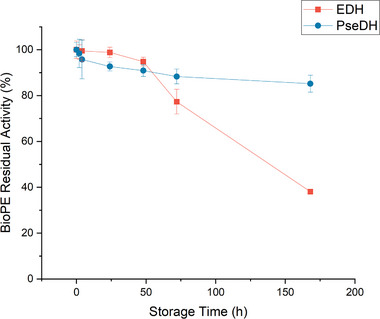
Effect of storage duration on the residual activity of the optimized BioPE with EDH and PsEDH. BioPE, bioactive Pickering emulsion; EDH, ephedrine dehydrogenase; PseDH, pseudoephedrine dehydrogenase.

The storage stability of the investigated BioPE systems at the settings for optimal functionality showed an equal or even higher stability of PseDH and EDH (Figure 2) compared to the free enzymes [14]. The catalytic activity of a BioPE with EDH decreased significantly after 48 h storage at 25°C, while the values for PseDH decreased slowly and steadily over 7 days storage at 5°C. A prolongation of stability was observed for EDH, which could be an effect of encapsulation [43, 44]. The chosen temperatures were based on the reported storage stability of the free enzymes, where EDH has a better half‐life time at 25°C (38.3 h) than at 5°C (2 h), whereas PseDH is more stable at 5°C (666 h).

The apparent improvement in the storage stability of EDH and PseDH in the BioPE can be attributed to the unique protective environment provided by the emulsion [21]. Encapsulation in a Pickering emulsion likely offers a microenvironment that shields the enzymes from direct exposure to harsh environments such as organic solvents [23]. This protective effect may be due to the physical barrier formed by the solid particles that stabilize the emulsion and encapsulate the enzymes, thus reducing their deactivation rate [17]. It is important to note that despite the presence of the surrounding solvent, which could potentially influence enzyme activity, the enzymes within the emulsion still exhibit activity. This suggests that the emulsion provides a sufficiently protective environment to sustain enzyme functionality even in the presence of surrounding solvents.

**FIGURE 3:**
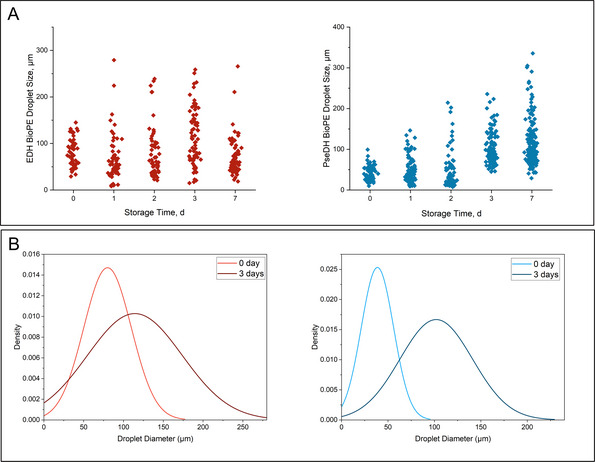
Influence of storage duration on droplet size and distribution in optimized BioPE with EDH and PseDH. (A) Scatter plot of droplet size as a function of storage duration. (B) Comparison of droplet size distribution in optimized BioPE with EDH and PsEDH at Days 0 and 3. BioPE, bioactive Pickering emulsion; EDH, ephedrine dehydrogenase; PseDH, pseudoephedrine dehydrogenase.

On the other hand, after storage under the above‐mentioned conditions, the BioPE showed significant changes in droplet size (Figure 3A) and droplet size distribution (Figure 3B) over time. In freshly prepared BioPE with EDH, the diameter of aqueous droplets in the cP was between 50 and 150 µm, while the size of droplets with PseDH was below 100 µm; the droplet size distribution was narrow in both preparations. After only 1 day of storage, droplets larger than 200 µm clearly appeared in the BioPE with EDH and droplets with diameters up to 150 µm were formed in the BioPE with PseDH. The trend was continuous over time with a maximum droplet size of about 250 µm in BioPE with EDH and even 350 µm in BioPE with PseEDH. After 3 days, the size distribution had widened considerably in both systems.

The observations on droplet size and size distribution changes are consistent with the destabilization processes typically observed in Pickering emulsions. The destabilization of Pickering emulsions can occur via a number of different mechanisms. The most widely demonstrated mechanism is coalescence, whereby emulsion droplets merge together [45, 46]. Another documented mechanism is Ostwald ripening, a process whereby larger particles grow at the expense of smaller ones in a dispersed system due to differences in solubility and surface energy, leading to the dissolution of smaller particles and their redeposition onto larger particles [47]. However, despite its critical importance to numerous processes and applications, the exact mechanism behind the destabilization of Pickering emulsions remains a subject of considerable debate [48].

It is noteworthy that no discernible correlation was observed between enzyme storage stability and the droplet size and size distribution in the BioPE systems that underwent the storage test. Despite the considerable alterations in droplet size and size distribution, particularly the widening of the distribution after 3 days of storage, the enzyme activity did not exhibit a parallel trend. For example, while the droplet size distribution of the BioPE with EDH exhibited a notable broadening by Day 3, its EDH activity had already decreased significantly by Day 2. In contrast, the BioPE with PseDH exhibited an earlier onset of droplet size broadening, beginning after just 1 day and resulting in a notable increase in droplet size and further widening of the distribution by Day 3. However, the PseDH activity in this system demonstrated a consistent decline throughout the entirety of the storage period. This observation indicates that the factors influencing emulsion destabilization may not directly correlate with enzyme activity and overall performance in this context, as is generally assumed. Further testing is necessary to confirm these findings, but today they suggest a potential decoupling of emulsion stability and enzyme performance that warrants further investigation.

## 4 Concluding Remarks

This study successfully demonstrates that the Pickering emulsion system can effectively facilitate the production of *R*‐PAC and *S*‐PAC using EDH and PseDH, respectively, and RjFDH for integrated cofactor regeneration. The findings confirm the viability and efficiency of the BioPE system for such enzyme cascade reactions. Furthermore, the optimization with EDH and PseDH resulted in notable performance metrics. A comparison with existing literature and patents reveals that the BioPE systems with EDH and PseDH demonstrate considerable competitiveness, even while using significantly lower enzyme concentrations. Moreover, the process has the potential to outperform current industrial processes, does not produce by‐products, and does not utilize harmful organic solvents or pressurized vessels. Although additional studies are still necessary, including up‐scaling substrate concentration, enzyme concentration, and reaction volume, this work represents a promising beginning in developing an alternative route for the production of *R*‐ and *S*‐PAC as well as related products.

## Conflicts of Interest

The authors declare no conflicts of interest.

## Supporting information



Supporting Information

## Data Availability

The data that support the findings of this study are available from the corresponding author upon reasonable request.
