# Supplementary material for: Reaction Engineering for Asymmetric R‐/S‐PAC Synthesis With Ephedrine or Pseudoephedrine Dehydrogenase in Pickering Emulsion
Source: Eng Life Sci. 2025 Jan 6;25(2):e202400069. doi: 10.1002/elsc.202400069 (PMC11842280; doi:10.1002/elsc.202400069)

**Supplementary Tables and Figures**

Table S1. Definitive Screening Experimental Design using BioPE with PseDH at a reaction temperature of 8 °C.

| Run | cP Ratio | Nanoparticle Concentration (g⋅L^-1^) | RjFDH Loading  (g⋅L^-1^) | PPD Concentration  (mol⋅L^-1^) | Dispersion Speed  (rpm) | Dispersion Time  (min) | Reaction Mixing Speed  (rpm) |
| --- | --- | --- | --- | --- | --- | --- | --- |
| 1 | 0.7 | 18.75 | 0.1 | 5 | 10000 | 1 | 20 |
| 2 | 0.6 | 50 | 0.25 | 8.75 | 10000 | 1 | 20 |
| 3 | 0.8 | 18.75 | 0.25 | 12.5 | 10000 | 1.5 | 20 |
| 4 | 0.8 | 50 | 0.1 | 12.5 | 10000 | 2 | 20 |
| 5 | 0.6 | 50 | 0.1 | 5 | 13750 | 2 | 20 |
| 6 | 0.8 | 50 | 0.25 | 5 | 17500 | 1 | 20 |
| 7 | 0.6 | 18.75 | 0.1 | 12.5 | 17500 | 1 | 20 |
| 8 | 0.8 | 18.75 | 0.175 | 5 | 17500 | 2 | 20 |
| 9 | 0.6 | 34.375 | 0.25 | 12.5 | 17500 | 2 | 20 |
| 10 | 0.6 | 18.75 | 0.25 | 5 | 10000 | 2 | 30 |
| 11 | 0.7 | 34.375 | 0.175 | 8.75 | 13750 | 1.5 | 30 |
| 12 | 0.8 | 50 | 0.1 | 12.5 | 17500 | 1 | 30 |
| 13 | 0.8 | 34.375 | 0.1 | 5 | 10000 | 1 | 40 |
| 14 | 0.6 | 50 | 0.175 | 12.5 | 10000 | 1 | 40 |
| 15 | 0.8 | 50 | 0.25 | 5 | 10000 | 2 | 40 |
| 16 | 0.6 | 18.75 | 0.1 | 12.5 | 10000 | 2 | 40 |
| 17 | 0.8 | 18.75 | 0.25 | 12.5 | 13750 | 1 | 40 |
| 18 | 0.6 | 18.75 | 0.25 | 5 | 17500 | 1 | 40 |
| 19 | 0.6 | 50 | 0.1 | 5 | 17500 | 1.5 | 40 |
| 20 | 0.8 | 18.75 | 0.1 | 8.75 | 17500 | 2 | 40 |
| 21 | 0.7 | 50 | 0.25 | 12.5 | 17500 | 2 | 40 |

Table S2. Follow-up Definitive Screening Experimental Design using BioPE with PseDH at a reaction temperature of 8 °C.

| Run | Nanoparticle Concentration (g⋅L^-1^) | Phosphate Buffer Concentration  (mol⋅L^-1^) | Potassium Formate Concentration  (mol⋅L^-1^) | Dispersion Speed  (rpm) | Dispersion Time  (min) |
| --- | --- | --- | --- | --- | --- |
| 1 | 50 | 0.55 | 1.5 | 17500 | 1 |
| 2 | 50 | 1 | 0.625 | 8000 | 1.5 |
| 3 | 18.75 | 0.55 | 0.625 | 8000 | 2 |
| 4 | 18.75 | 1 | 0.625 | 17500 | 2 |
| 5 | 50 | 0.1 | 1.5 | 8000 | 1 |
| 6 | 34.375 | 0.55 | 1.0625 | 12750 | 1.5 |
| 7 | 18.75 | 1 | 1.0625 | 8000 | 1 |
| 8 | 50 | 1 | 1.5 | 8000 | 2 |
| 9 | 18.75 | 0.1 | 1.5 | 17500 | 1.5 |
| 10 | 18.75 | 0.1 | 1.5 | 8000 | 2 |
| 11 | 50 | 0.1 | 1.0625 | 17500 | 2 |
| 12 | 18.75 | 0.1 | 0.625 | 17500 | 1 |
| 13 | 50 | 1 | 0.625 | 17500 | 1 |
| 14 | 34.375 | 0.1 | 0.625 | 8000 | 1 |
| 15 | 34.375 | 1 | 1.5 | 17500 | 2 |
| 16 | 18.75 | 1 | 1.5 | 12750 | 1 |
| 17 | 50 | 0.1 | 0.625 | 12750 | 2 |

Table S3. Experimental Design for BioPE with EDH at a reaction temperature of 25 °C.

| Pattern | Run | cP Ratio | PPD Concentration (mmol⋅L^-1^) | Phosphate Buffer Concentration  (mol⋅L^-1^) | Potassium Formate Concentration  (mol⋅L^-1^) | RjFDH Loading  (g⋅L^-1^) |
| --- | --- | --- | --- | --- | --- | --- |
| +−+++ | 1 | 0.7500 | 12.5 | 0.50 | 1.500 | 0.80 |
| 00000 | 2 | 0.6375 | 37.5 | 0.25 | 0.875 | 0.50 |
| 00a00 | 3 | 0.6375 | 37.5 | 0.00 | 0.875 | 0.50 |
| −−−++ | 4 | 0.5250 | 12.5 | 0.00 | 1.500 | 0.80 |
| 0000A | 5 | 0.6375 | 37.5 | 0.25 | 0.875 | 1.05 |
| −−++− | 6 | 0.525 | 12.5 | 0.50 | 1.500 | 0.20 |
| +−−−+ | 7 | 0.7500 | 12.5 | 0.00 | 0.250 | 0.80 |
| 00A00 | 8 | 0.6375 | 37.5 | 0.71 | 0.875 | 0.50 |
| −++−− | 9 | 0.5250 | 62.5 | 0.5 | 0.250 | 0.20 |
| −+−−+ | 10 | 0.5250 | 62.5 | 0.00 | 0.250 | 0.80 |
| 00000 | 11 | 0.6375 | 37.5 | 0.25 | 0.875 | 0.50 |
| ++++− | 12 | 0.7500 | 62.5 | 0.50 | 1.500 | 0.20 |
| 00000 | 13 | 0.6375 | 37.5 | 0.25 | 0.875 | 0.50 |
| −−−−− | 14 | 0.5250 | 12.5 | 0.00 | 0.250 | 0.20 |
| 00000 | 15 | 0.6375 | 37.5 | 0.25 | 0.875 | 0.50 |
| 0a000 | 16 | 0.6375 | 12.5 | 0.25 | 0.875 | 0.50 |
| +−+−− | 17 | 0.7500 | 12.5 | 0.50 | 0.25 | 0.20 |
| −−+−+ | 18 | 0.5250 | 12.5 | 0.50 | 0.25 | 0.80 |
| +++−+ | 19 | 0.7500 | 62.5 | 0.50 | 0.25 | 0.80 |
| ++−−− | 20 | 0.7500 | 62.5 | 0.00 | 0.25 | 0.20 |
| 0000a | 21 | 0.6375 | 37.5 | 0.25 | 0.875 | 0.04 |
| 000A0 | 22 | 0.6375 | 37.5 | 0.25 | 2.013 | 0.50 |
| ++−++ | 23 | 0.7500 | 62.5 | 0.00 | 1.500 | 0.80 |
| −+−+− | 24 | 0.5250 | 62.5 | 0.00 | 1.500 | 0.20 |
| 00000 | 25 | 0.6375 | 37.5 | 0.25 | 0.875 | 0.50 |
| 0A000 | 26 | 0.6375 | 83.0 | 0.25 | 0.875 | 0.50 |
| 00000 | 27 | 0.6375 | 37.5 | 0.25 | 0.875 | 0.50 |
| 000a0 | 28 | 0.6375 | 37.5 | 0.25 | 0.063 | 0.50 |
| A0000 | 29 | 0.8000 | 37.5 | 0.25 | 0.875 | 0.50 |
| a0000 | 30 | 0.4750 | 37.5 | 0.25 | 0.875 | 0.50 |
| −++++ | 31 | 0.5250 | 62.5 | 0.50 | 1.500 | 0.80 |
| +−−+− | 32 | 0.7500 | 12.5 | 0.00 | 1.500 | 0.20 |

Table S4. Experimental Design for for BioPE with PseDH at a reaction temperature of 8 °C.

| Pattern | Run | cP Ratio | PPD Concentration  (mmol⋅L^-1^) | Potassium Phosphate Buffer Concentration  (mol⋅L^-1^) | Potassium Formate Concentration  (mol⋅L^-1^) | RjFDH Loading  (g⋅L^-1^) |
| --- | --- | --- | --- | --- | --- | --- |
| +++−+ | 1 | 0.7500 | 42.5 | 1.00 | 0.100 | 0.80 |
| 00a00 | 2 | 0.6375 | 30 | 0.00 | 0.800 | 0.50 |
| 00000 | 3 | 0.6375 | 30 | 0.55 | 0.800 | 0.50 |
| −−+−+ | 4 | 0.5250 | 17.5 | 1.00 | 0.100 | 0.80 |
| ++++− | 5 | 0.7500 | 42.5 | 1.00 | 1.500 | 0.20 |
| −+−−+ | 6 | 0.5250 | 42.5 | 0.10 | 0.100 | 0.80 |
| 0A000 | 7 | 0.6375 | 59.4 | 0.55 | 0.800 | 0.50 |
| ++−++ | 8 | 0.7500 | 42.5 | 0.10 | 1.500 | 0.80 |
| 00000 | 9 | 0.6375 | 30 | 0.55 | 0.800 | 0.50 |
| +−+−− | 10 | 0.7500 | 17.5 | 1.00 | 0.100 | 0.20 |
| 00000 | 11 | 0.6375 | 30 | 0.55 | 0.800 | 0.50 |
| 0000A | 12 | 0.6375 | 30 | 0.55 | 0.800 | 1.21 |
| 00A00 | 13 | 0.6375 | 30 | 1.33 | 0.800 | 0.50 |
| A0000 | 14 | 0.8000 | 30 | 0.55 | 0.800 | 0.50 |
| 0a000 | 15 | 0.6375 | 13.3 | 0.55 | 0.800 | 0.50 |
| 0000a | 16 | 0.6375 | 30 | 0.55 | 0.800 | 0.04 |
| −−−−− | 17 | 0.5250 | 17.5 | 0.10 | 0.100 | 0.20 |
| 000a0 | 18 | 0.6375 | 30 | 0.55 | 0.050 | 0.50 |
| +−−−+ | 19 | 0.7500 | 17.5 | 0.10 | 0.100 | 0.80 |
| −+−+− | 20 | 0.5250 | 42.5 | 0.10 | 1.500 | 0.20 |
| a0000 | 21 | 0.5000 | 30 | 0.55 | 0.800 | 0.50 |
| 00000 | 22 | 0.6375 | 30 | 0.55 | 0.800 | 0.50 |
| 00000 | 23 | 0.6375 | 30 | 0.55 | 0.800 | 0.50 |
| 00000 | 24 | 0.6375 | 30 | 0.55 | 0.800 | 0.50 |
| 000A0 | 25 | 0.6375 | 30 | 0.55 | 2.448 | 0.50 |
| +−−+− | 26 | 0.7500 | 17.5 | 0.10 | 1.500 | 0.20 |
| ++−−− | 27 | 0.7500 | 42.5 | 0.10 | 0.100 | 0.20 |
| −++−− | 28 | 0.5250 | 42.5 | 1.00 | 0.100 | 0.20 |
| +−+++ | 29 | 0.7500 | 17.5 | 1.00 | 1.500 | 0.80 |
| −++++ | 30 | 0.5250 | 42.5 | 1.00 | 1.500 | 0.80 |
| −−++− | 31 | 0.5250 | 17.5 | 1.00 | 1.500 | 0.20 |
| −−−++ | 32 | 0.5250 | 17.5 | 0.10 | 1.500 | 0.80 |

Figure S1. Space-time yield of BioPE with PseDH at reaction temperatures of 8 °C and 25 °C.


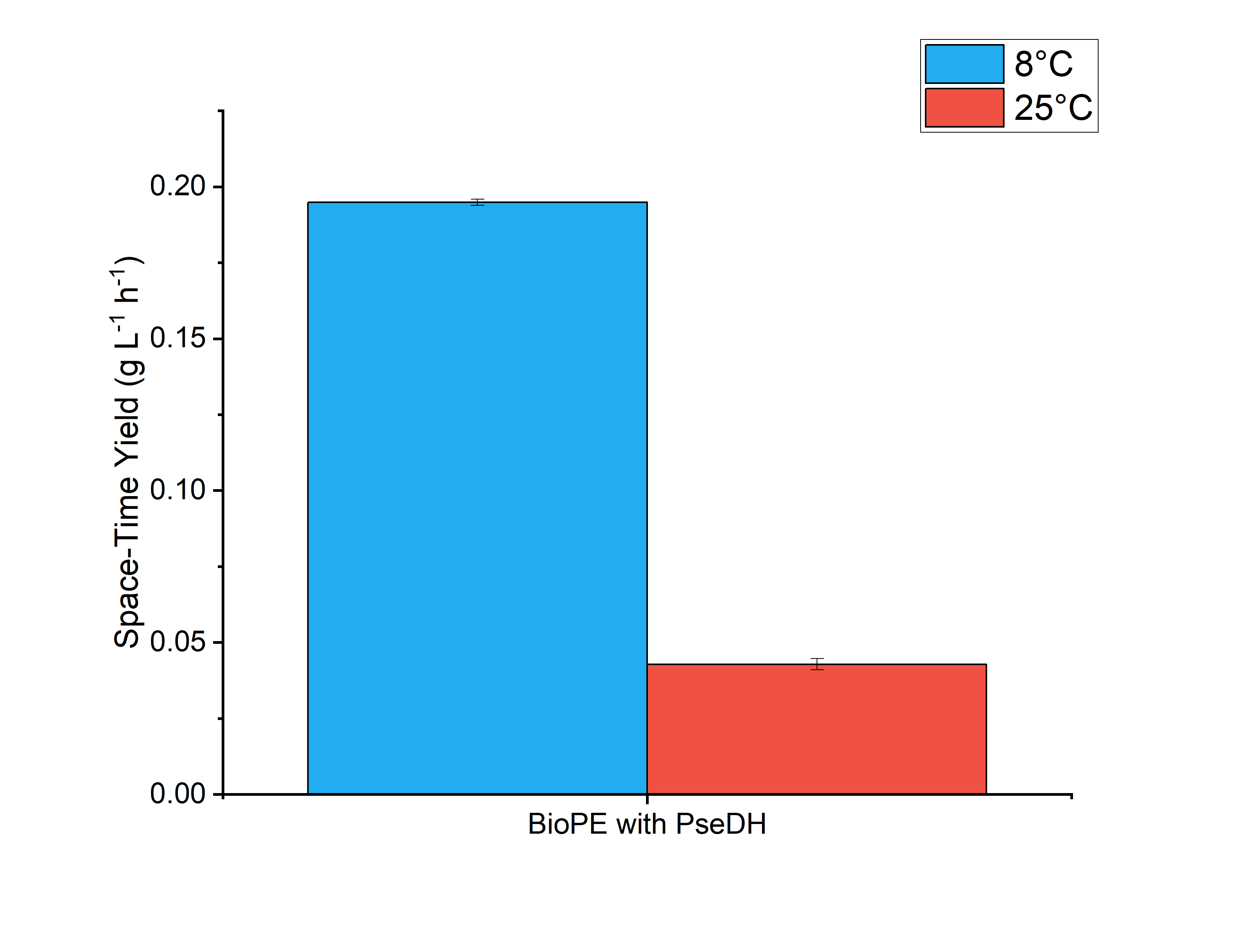


Figure S2. Sample microscopic images of BioPE with EDH at day 0 (left) and day 3 (right).


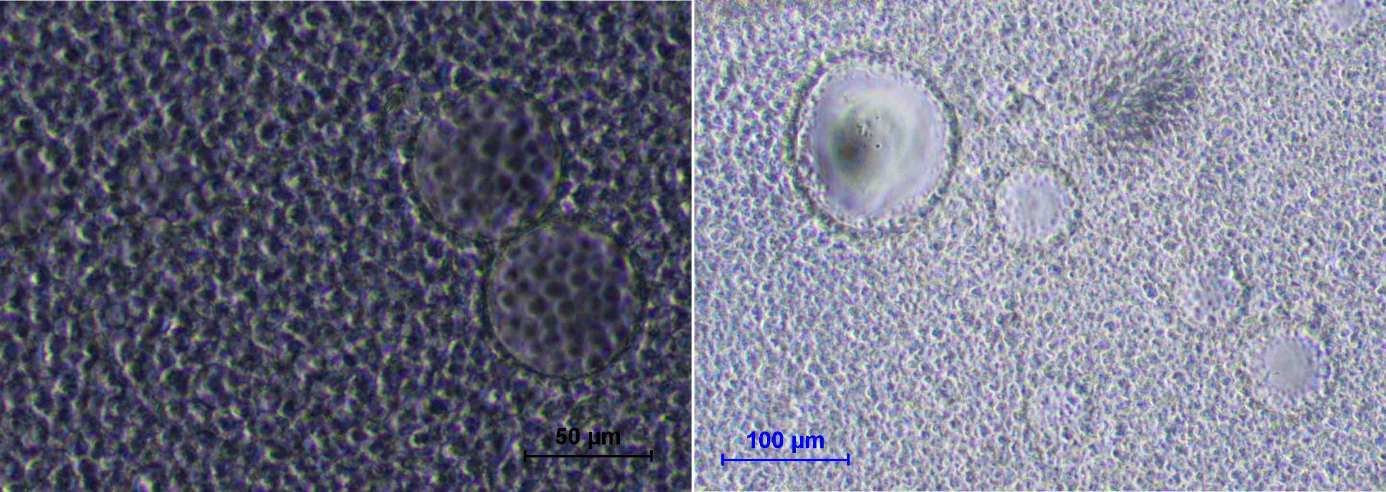


Figure S3. Sample microscopic images of BioPE with PseDH at day 0 (left) and day 3 (right).


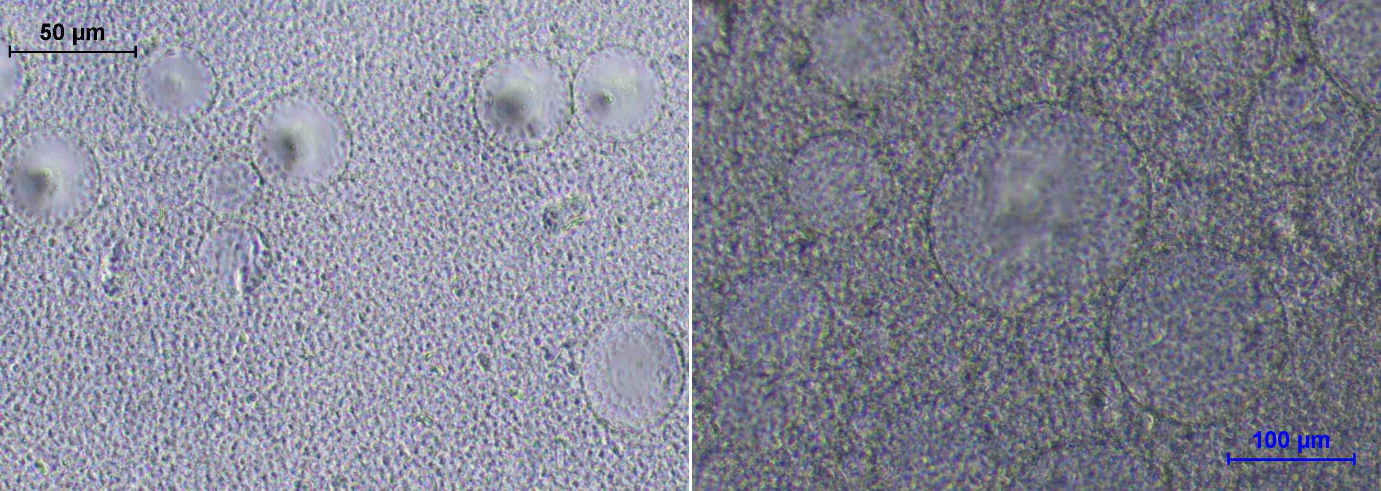

Supplement: Supplementary file 1 — Supporting Information [file ELSC-25-e202400069-s001.docx]
